# Double-stranded DNA donors and CRISPR-Cas9 for universal correction of mutations causing cystic fibrosis in human airway cells

**DOI:** 10.1016/j.omtn.2026.103049

**Published:** 2026-08-03

**Authors:** Vrishti Sinha, Paul G. Ayoub, Colin J. Juett, Lindsay E. Lathrop, Ruth A. Foley, Ruby A. Sims, Joseph D. Long, Emily C. Duggan, Neil R. Fernandes, Beate Illek, Brigitte N. Gomperts, Steven J. Jonas, Donald B. Kohn

**Affiliations:** 1 Department of Human Genetics, University of California, Los Angeles, Los Angeles, CA 90095, USA; 2 Molecular Biology Institute, University of California, Los Angeles, Los Angeles, CA 90095, USA; 3 Department of Microbiology, Immunology, and Molecular Genetics, University of California, Los Angeles, Los Angeles, CA 90095, USA; 4 Department of Bioengineering, University of California, Los Angeles, Los Angeles, CA 90095, USA; 5 Department of Pediatrics, David Geffen School of Medicine, University of California, Los Angeles, Los Angeles, CA 90095, USA; 6 Department of Pediatrics, School of Medicine, University of California, San Diego, San Diego, CA 92103, USA; 7 Jonsson Comprehensive Cancer Center, University of California, Los Angeles, Los Angeles, CA 90095, USA; 8 Eli & Edythe Broad Center of Regenerative Medicine and Stem Cell Research, University of California, Los Angeles, Los Angeles, CA 90095, USA; 9 California NanoSystems Institute, University of California, Los Angeles, Los Angeles, CA 90095, USA

**Keywords:** MT: RNA/DNA editing, cystic fibrosis, site-specific insertion, nonviral gene delivery, homology-directed repair, cystic fibrosis transmembrane conductance regulator, *CFTR*

## Abstract

Cystic fibrosis (CF) is a devastating genetic disease caused by mutations in the *cystic fibrosis transmembrane conductance regulator* (*CFTR*) gene. As morbidity and mortality from CF results from a lack of mucus clearance that leads to chronic bacterial infections and progressive loss of lung function, site-specific insertion of a *CFTR* cDNA into the endogenous *CFTR* locus in airway basal stem cells (ABSCs) could prove curative for all disease-causing mutations. This study describes the development of genome-editing approach utilizing nonviral reagents, designed to be packaged into nonviral delivery systems. An sgRNA targeting the 5′ untranslated region (UTR) of *CFTR* was characterized as directing high on-target cutting and displaying a safe off-target profile. Airway cell lines electroporated with chemically modified (1-Aminohexane, AmC6), linear double-stranded DNA (ldsDNA) constructs were utilized as a homology directed repair (HDR) donor, initially optimized with an mCitrine reporter. Expectedly, when the 780 bp mCitrine cDNA was replaced with the 4.4 kb *CFTR* cDNA, integration efficiency dropped significantly. However, 1%–2% integration of codon-optimized donors was sufficient to restore CFTR expression in the bulk-edited population of a human bronchial epithelial cell line, 16HBE14o- (16HBE), to levels reaching 50% of wild-type expression as measured by western blot. Electrophysiological validation of CFTR ion channel function measured via Ussing chamber assay revealed that these bulk-edited populations exhibit greater than 40% restoration of the chloride ion currents of the measured wild-type controls. These results demonstrate that low levels of *CFTR* integration can be made therapeutically relevant by optimizing the designs of gene editing reagents. Importantly, this work utilizes nonviral-editing reagents, an essential step toward *in vivo* gene therapy for CF.

## Graphical abstract

**Figure:**
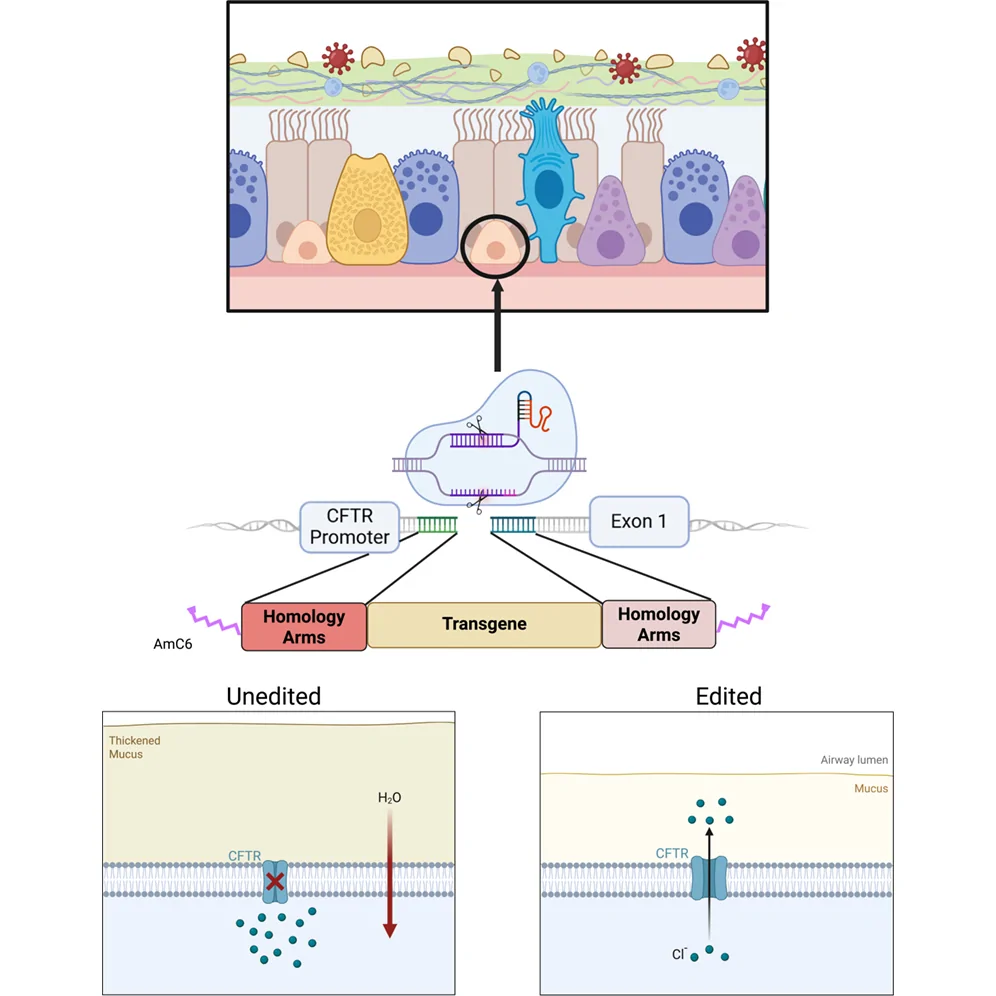

## Teaser

This study presents a universal gene-editing strategy for the correction of cystic fibrosis mutations via site-specific integration of a linear double-stranded DNA (ldsDNA) into the endogenous locus utilizing CRISPR-Cas9 reagents. This strategy demonstrates successful functional protein restoration in an immortalized airway epithelial cell line despite overall low editing rates.

## Introduction

Cystic fibrosis (CF) is a rare autosomal recessive monogenic disorder resulting from mutations in the *cystic fibrosis transmembrane conductance regulator* (*CFTR*) gene which encodes for an epithelial ion channel protein that transports chloride and bicarbonate across the membrane.^1^^,^^2^ This transmembrane protein is essential for maintaining proper homeostatic osmosis in the epithelial layers of the lungs, pancreas, bile ducts, intestines, and sweat glands.^2^ When mutated, dysfunctional CFTR protein fails to transport chloride across these various epithelia properly such that the mucus layer dehydrates, leading to frequent pulmonary infections and inflammation that ultimately results in progressive lung destruction and respiratory failure.^3^ Despite affecting multiple organs, patient morbidity and mortality result primarily from complications in the airway, thereby highlighting the importance of targeting the lungs for corrective gene therapy approaches.^4^

The use of both corrector and potentiator modulators to improve either the folding or function of the mutated CFTR protein, respectively, has greatly improved the health of many CF patients.^5^^,^^6^^,^^7^ However, despite the success of modulator therapies, not all patients are able to benefit from these medications, particularly those with null mutations or very low levels of CFTR expression and those who develop side effects from these treatments. Roughly 10% of patients have mutations within these categories, highlighting the need for alternative therapies to maximize the benefits for the entire CF patient population.^5^^,^^8^

The genome-editing capabilities enabled by the CRISPR-Cas9 system have allowed for site-specific repair or replacement of mutated genes in a wide variety of disease contexts. Consequently, for *CFTR*, stable integration of a normal *CFTR* cDNA into the endogenous *CFTR* locus in airway basal stem cells (ABSCs) downstream of the promoter has proven beneficial to restoring expression of functional CFTR levels.^9^^,^^10^ Since this strategy is mutation agnostic, it represents a universal approach that can be functionally curative for all CF patients, regardless of the mutation type.

The limiting factor of this site-specific integration approach is the large size of the coding region of the *CFTR* gene. At roughly 4.4 kb, traditional homology directed repair (HDR) donors carrying the *CFTR* coding sequence exceed the packaging capacity of a single adeno-associated virus type 6 (AAV6). As a result, traditional AAV-based HDR approaches have shown low efficiency in delivering the necessary coding sequence.^11^^,^^12^ Some groups have also targeted high frequency mutations with base and prime editing, achieving partial functional correction of the CFTR protein.^13^^,^^14^^,^^15^ Still, with over 2,000 known *CFTR* variants of varying pathogenic significance, these strategies will leave many patients without a curative gene therapeutic option.^16^ Novel strategies to address the size limitation have emerged by generating shortened *CFTR* variants that package into a single AAV6 vector^17^ or by delivering the full-length coding sequence of *CFTR* in segments using AAV6 vectors.^18^ In the latter approach, edited airway basal cells were enriched *ex vivo* and retained their phenotype, restoring CFTR function to near wild-type cells as measured *via* Ussing chamber assay.^19^^,^^20^

**Figure 1:**
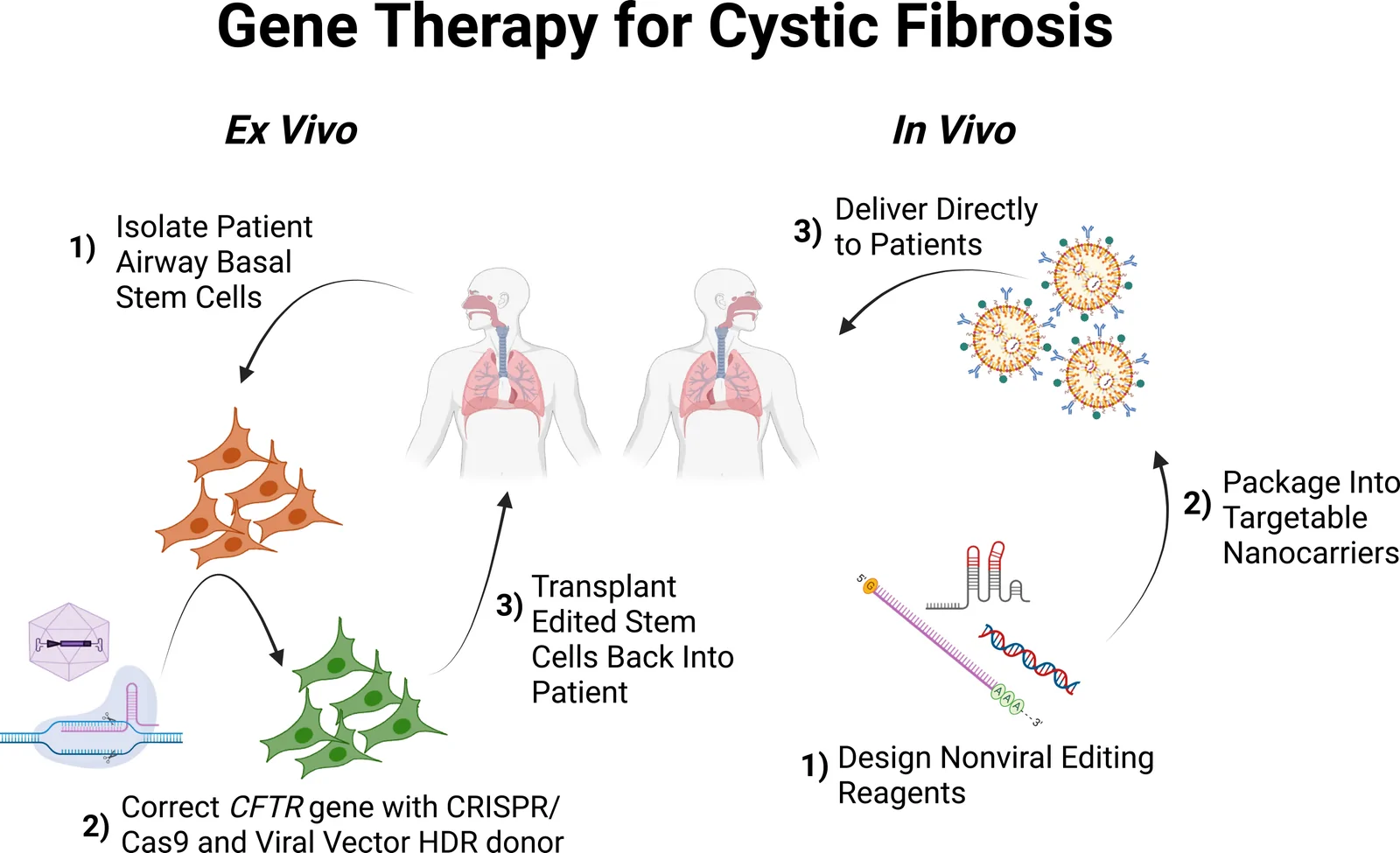
Gene therapy for cystic fibrosis Description of two gene therapy approaches for cystic fibrosis achieved via *ex vivo* or *in vivo* genome editing. Created with BioRender.

Still, the necessity of both enrichment of edited cells and the use of AAV6 as an HDR donor requires the editing to be performed *ex vivo* (Figure 1). Apart from editing efficiencies concerning large donors, the process of autologous transplant for airway basal cells is limited by complex roadblocks that have not yet been overcome.^21^^,^^22^ Alternatively, *in vivo* approaches could rely on the nonviral delivery of genome editing reagents directly to the patient’s lung using lipid nanoparticle (LNP)-based carriers, thereby potentially overcoming the limitations of autologous transplantation (Figure 1). Furthermore, this *in vivo* editing approach would be less invasive to the patient and avoid the potential innate immune response that may occur in response to viral vectors.

Due to these benefits, we sought to develop a genome editing system using nonviral reagents to insert the *CFTR* cDNA downstream of the endogenous *CFTR* promoter that could be optimized for *in vivo* delivery. We aimed to override the majority of coding region mutations and acquire appropriate expression of CFTR to reduce disease phenotype, which is expected to be achievable with a population of about 10% CFTR-expressing cells.^23^ Here, we report an editing system that utilized CRISPR-Cas9 to insert a codon-optimized *CFTR* cDNA cassette into the 5′ untranslated region (UTR) of the *CFTR* gene. Using this system, CFTR protein was successfully produced in a CFTR-null bronchial epithelial cell line even at low (1%–2%) integration efficiencies. When edited cell populations were grown as submerged cell monolayers in Transwell membrane inserts, and assessed for functional restoration, chloride currents of up to 80% that of wild type were achieved. These results demonstrate a step toward a mutation agnostic nonviral gene-editing approach to stably treat CF.

## Results

**Figure 2:**
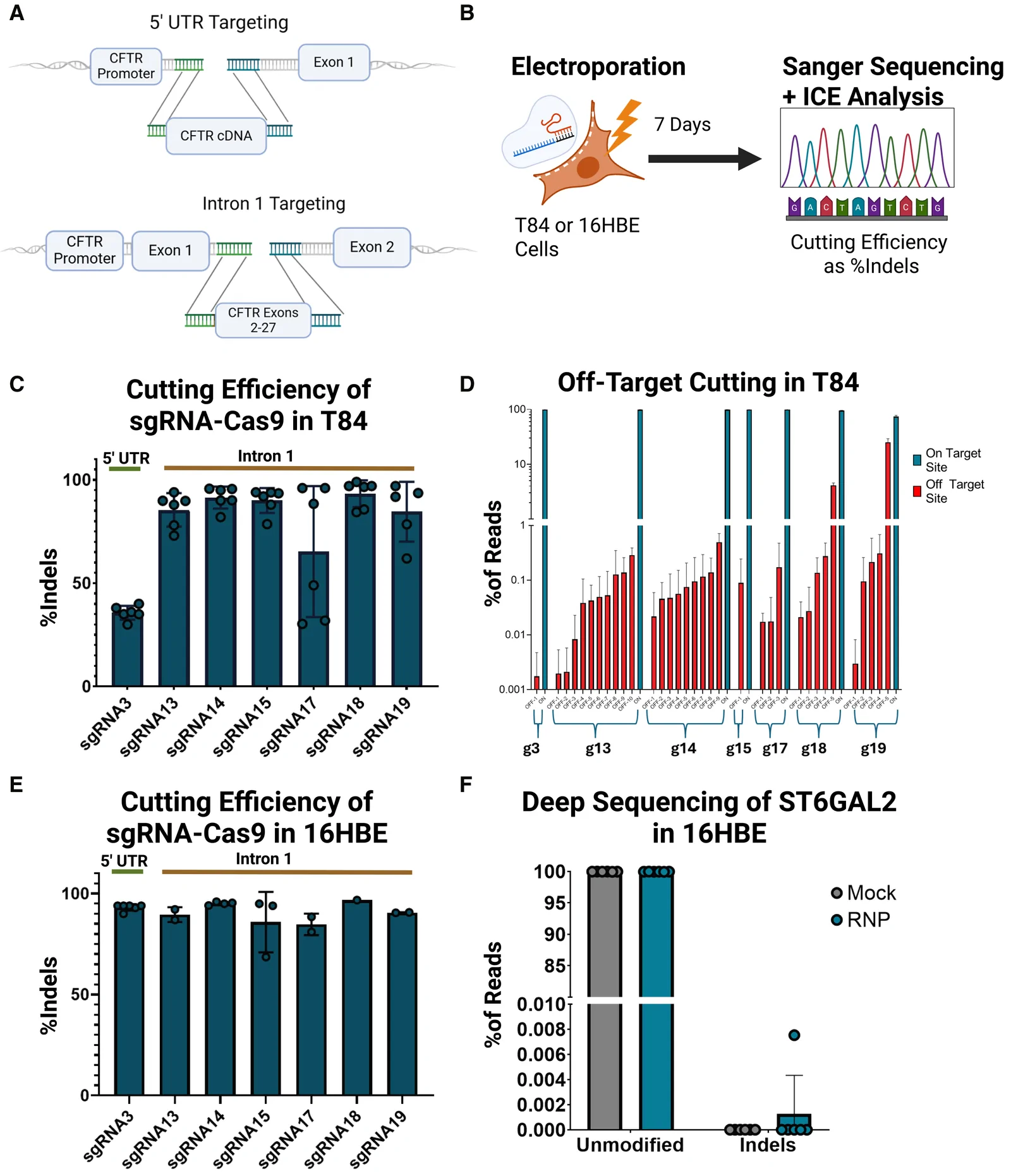
Characterizing sgRNA targeting *CFTR* locus (A) Schematic demonstration of two editing strategies based on two sgRNA targets. (B) Method timeline for screening sgRNA-mediated cutting efficiency. (C) Cutting efficiency, measured by % indels, in T84 cells (*n* = 6). Each dot represents a different electroporated replicate. (D) Percent of total reads that are on-target (blue) or off-target (red) from the highest dose of dsODN delivered (5 μM) in GUIDE-seq analysis (*n* = 3). Different red bars represent distinct sites. (E) Cutting efficiency, measured by % indels, in 16HBE cells (*n* = 2–6). Each dot represents a different electroporated replicate. (F) Results from deep sequencing at nominated off-target site, *ST6GAL2*, in 16HBEs edited with sgRNA3 (*n* = 6). Statistical significance was analyzed using a one-way ANOVA followed by multiple paired comparisons for normally distributed data (Tukey test). All statistical tests were two-tailed and ns *p* value of >0.05, ∗*p* < 0.05, ∗∗*p* < 0.01, ∗∗∗*p* < 0.001, ∗∗∗∗*p* < 0.0001. (A) and (B) are created with BioRender.

We began our studies by investigating the optimal site in the endogenous *CFTR* locus to insert a *CFTR* cDNA cassette. As such, various single guide RNAs (sgRNA) targeting either the 5′UTR or intron 1 of the *CFTR* gene were screened for frequency of target site disruption (Figure 2A). Initially, guide screening was performed in a T84 cell line derived from a human lung metastasis of colon carcinoma. Screening was repeated in the 16HBE cell line, which is more conducive for downstream functional assays and therefore a more clinically relevant model than T84 cells (Figure 2B). All guides (Table S1) were screened with the addition of the Alt-R Cas9 electroporation enhancer to assist in increasing *ex vivo*-editing efficiency, thereby achieving a 2-fold increase in CRISPR-Cas9 cutting efficiency (S1A and S1B). In edited T84 cells, sgRNA 3 targeting the 5′UTR and delivered as sgRNA-Cas9 ribonucleoprotein (RNP) had an average cutting frequency of 35.7% as measured by the formation of insertion-deletions (indels) (Figure 2C). In comparison, the intron 1 guides (sgRNA13-19) had average cutting efficiencies ranging from 65% up to 93.3% (Figure 2C).

The off-target activity of sgRNA-directed Cas9 was assessed *via* genome-wide, unbiased identification of DSBs enabled by sequencing (GUIDE-seq) in the T84 cell line.^24^^,^^25^ Although off-target reads were observed from all screened guides, the 5′UTR sgRNA 3 and the intron 1 sgRNA 15 had the fewest measured off-target cut sites, and these were rare with all comprising less than 0.1% of total reads (Figure 2D).

In follow-up guide screening studies in the more clinically relevant 16HBE cells, the cutting efficiency of sgRNA 3 dramatically increased to 93.2% compared to its lower activity seen in the T84s, while the intron 1 guide maintained high cutting efficiency in both cell lines, with up to 96.8% indels in the 16HBE cells (Figure 2E). This result highlights the differences in guide efficiency across cell lines.

The off-target activity of sgRNA 3 in 16HBE cells was analyzed via GUIDE-seq, which revealed a single off-target site into intron 1 of *ST6GAL2*, a gene encoding for a sialoglycosyltransferase involved in myoblast proliferation^26^ (S1C). The off-target site is 22.5 kb from the first coding exon of the gene, but 50 bp away from an ENCODE4 cis-regulatory element candidate (EH38E3363205). Deep sequencing at this site revealed indels in less than 0.01% of reads (Figure 2F; S1D). Due to the increased safety profile of sgRNA 3 and its potential to treat mutations found within exon 1 (which would not be addressed with the downstream intron 1 sgRNA), sgRNA 3 was selected to test HDR donor reagents since the entire *CFTR* cDNA could be inserted into the 5′UTR.

**Figure 3:**
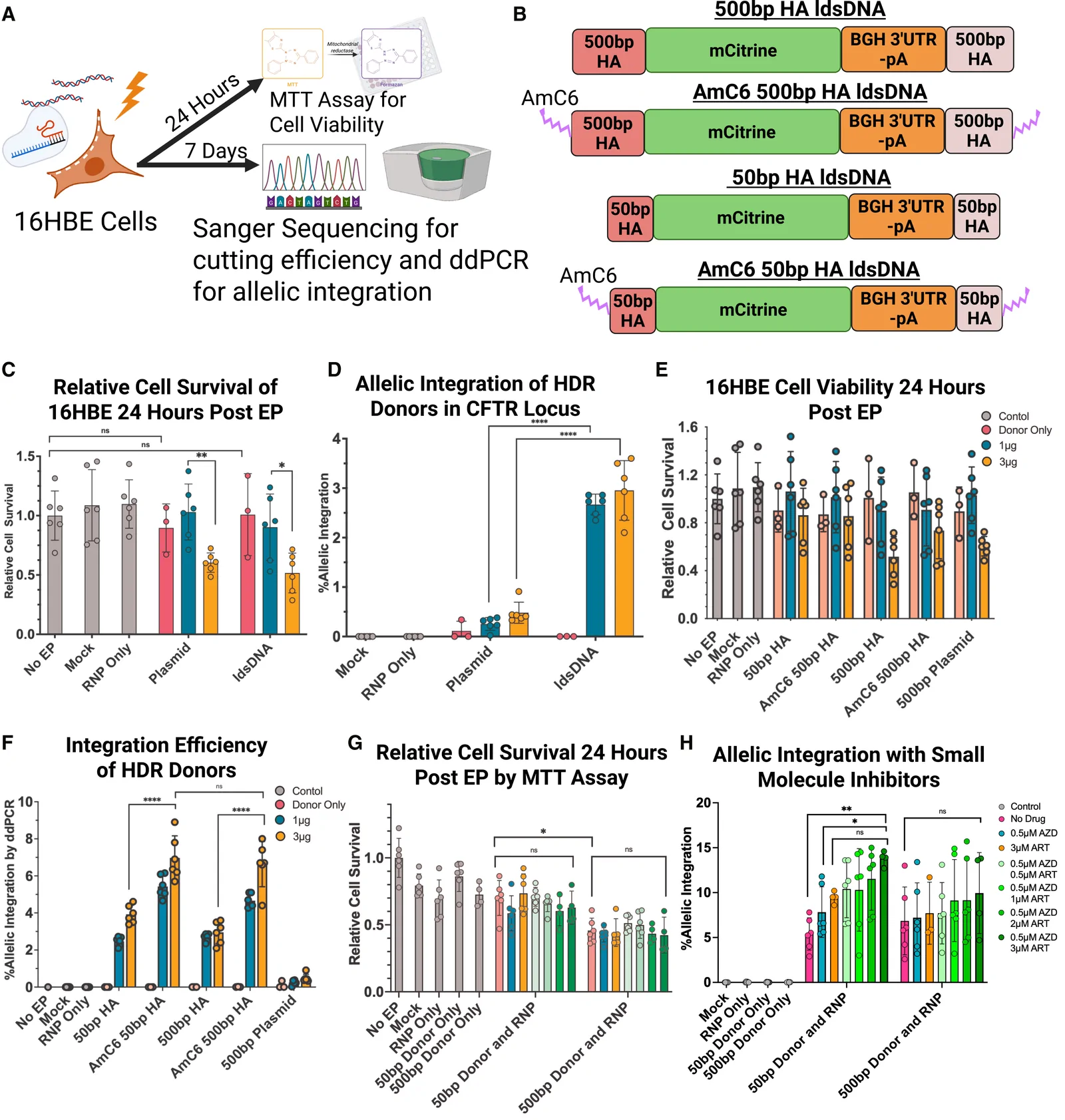
Optimization of nonviral HDR donor-editing reagents (A) Illustration of workflow for screening sgRNA-mediated cutting efficiency. Cells were divided immediately following electroporation to perform MTT assay and molecular analysis separately. (B) Schematics for mCitrine HDR donors. All donors contain mCitrine gene and 3′UTR of the bovine growth hormone gene. Donors varied based on homology arm size (50 bp or 500 bp) and inclusion or exclusion of AmC6 modification. (C and D) (C) Relative cell survival and (D) integration after electroporation delivering plasmid or linear dsDNA HDR donor with 500 bp HA construct to 16HBE cells (*n* = 3). (E and F) (E) Relative cell survival and (F) integration after electroporation delivering 50 bp homology arm donors with or without AmC6 and 500 bp donors with or without AmC6 (*n* = 3). (G and H) (G) Relative cell survival and (H) integration after electroporation delivering AmC6 50 bp HA and AmC6 500 bp HA donors with varied concentrations of AZD7648 and ART558 in recovery media (*n* = 3). Each dot represents a different electroporated replicate. (F) Results from GUIDE-seq analysis of sgRNA3 in 16HBEs from the highest dose of dsODN delivered (5 μM) in GUIDE-seq analysis (*n* = 3). Data are represented as mean ± SD of biological triplicates from three experiments. Statistical significance was analyzed using a one-way ANOVA followed by multiple paired comparisons for normally distributed data (Tukey test). All statistical tests were two-tailed and ns *p* value of >0.05, ∗*p* < 0.05, ∗∗*p* < 0.01, ∗∗∗*p* < 0.001, ∗∗∗∗*p* < 0.0001. (A) and (B) are created with BioRender.

Next, nonviral DNA constructs were tested as HDR donors. To first assess donor integration potential, an mCitrine construct was designed and tested to target the 5′UTR cut site of sgRNA 3 with flanking homology arms (HA) of roughly 500 bps. To improve expression, bovine growth hormone (BGH) polyA signal sequence was included in the reporter construct as well. In 16HBEs, this construct was delivered as either plasmid forms or as ldsDNA HDR donors. The safety and efficacy of these donors were assessed by delivery combined with sgRNA3-Cas9 RNP, followed by a 3-(4,5-dimethylthiazol-2-yl)-2,5-diphenyltetrazolium bromide (MTT) Assay^27^ to determine relative cell viability, and an in-out digital droplet PCR (ddPCR) assay on genomic DNA of edited cells to determine allelic integration frequencies (Figures 3A and 3B). Specificity and accuracy of the ddPCR design was compared to flow cytometry (S2A). As expected, the flow cytometry data demonstrated that the percentage of mCitrine^+^ cells was twice that as the percentage of allelic integration, indicating predominantly single-allele integration (S2B).

While delivery of the donors on their own did not significantly affect cell viability, considerable toxicity was observed in the presence of RNP in combination with the highest doses of both ldsDNA and plasmid HDR donors (Figure 3C). Despite similar toxicity profiles, the ldsDNA dramatically outperformed plasmid in allelic integration frequency, with over 6-fold greater integration (Figure 3D).

Nanoplasmids were also tested for HDR donor delivery in T84 cells and demonstrated significantly less toxicity than the plasmid HDR donor (S3A). However, the nanoplasmid exhibited lower integration efficiencies compared to ldsDNA, with no significant difference compared to the plasmid HDR donor (S3B).

It is well established that HDR donor size inversely correlates with integration efficiency.^28^^,^^29^ The ldsDNA donor encoding for the mCitrine cassette, which is roughly 2.0 kb, achieved 3.0% integration. In contrast, the *CFTR* cDNA alone, without necessary homology arms or regulatory elements, is roughly 4.4 kb. Given our aim to reach the therapeutic threshold of 6%–10% targeted integration, optimization of modifications and reagents to boost transgene integration was necessary prior to testing donors containing the *CFTR* cDNA.

To enhance HDR efficiency, the initial ldsDNA donor was designed with larger 500 bp homology arms that included the *CFTR* promoter sequence on the 5′ end. However, inclusion of the promoter sequence raised concerns about potential dysregulation of nearby genes due to off-target integration events or random, nonspecific trapping of the donor. To mitigate this risk, we designed and tested HDR donors with shorter 50 bp homology arms that excluded the *CFTR* promoter sequence and evaluated whether efficient integration could still be achieved.

Furthermore, chemical modifications on the 5′ ends of ldsDNA donors, such as 1-Aminohexane (AmC6), have been shown to boost gene knock-in rates up to 5-fold and permit the use of shorter homology arm lengths with minimal loss of integration efficiency (Figure 3B).^30^ We sought to assess the effect of AmC6 modifications on the 5′ ends of the donor construct in combination with the reduced homology arm length.

In edited 16HBE cells, the relative cell survival was unchanged with the addition of the AmC6 modification (Figure 3E). Encouragingly, the allelic integration increased approximately 2-fold with the AmC6 modification across both 50 bp and 500 bp donor homology arms. Furthermore, reduction in the size of the homology arms did not significantly change the integration rate, supporting the use of the 50 bp homology arm HDR donor (Figure 3F). Notably, in the T84 cell line, the addition of AmC6 to the donors had a modest increase in integration. Moreover, reducing homology arm length in this context decreased knock-in efficiency (S3C and S3D). These findings suggest the benefit of the AmC6 modification might be cell-type specific.

To support efforts to increase site-specific integration of transgenes, there have been several recent studies into small molecule inhibitors acting on the DNA-dependent protein kinase catalytic subunit (DNA-PKc) (AZD7648, AZD) and Polymerase Theta (ART558, ART), two important mediators of the non-homologous end joining (NHEJ) and microhomology-mediated end joining (MMEJ) pathways, respectively.^31^^,^^32^ To clarify whether the 500 bp donor might have an improved propensity for HDR, both the 50 bp and 500 bp HA donors with the AmC6 modification were tested head-to-head in a drug titration. Cell survival was unaffected by the addition of the drugs at any concentration or combination, but the 50 bp HA donor supported significantly better cell survival than the 500 bp HA donor (Figure 3G).

Surprisingly, the 500 bp HA donor had no significant differences in integration with the addition of the inhibitors. However, the 50 bp HA donor had significantly improved integration, doubling to an average of 13.80% knock-in efficiency at the highest combined dose of AZD and ART (Figure 3H). Moreover, the combination of the drugs supported significantly higher integration than AZD on its own (Figure 3H). From conducting these optimization experiments, the optimal candidate ldsDNA donor included 50 bp homology arms, AmC6 modifications on the 5′ ends, and the addition of 0.5 μM AZD and 3 μM ART into the recovery medium.

**Figure 4:**
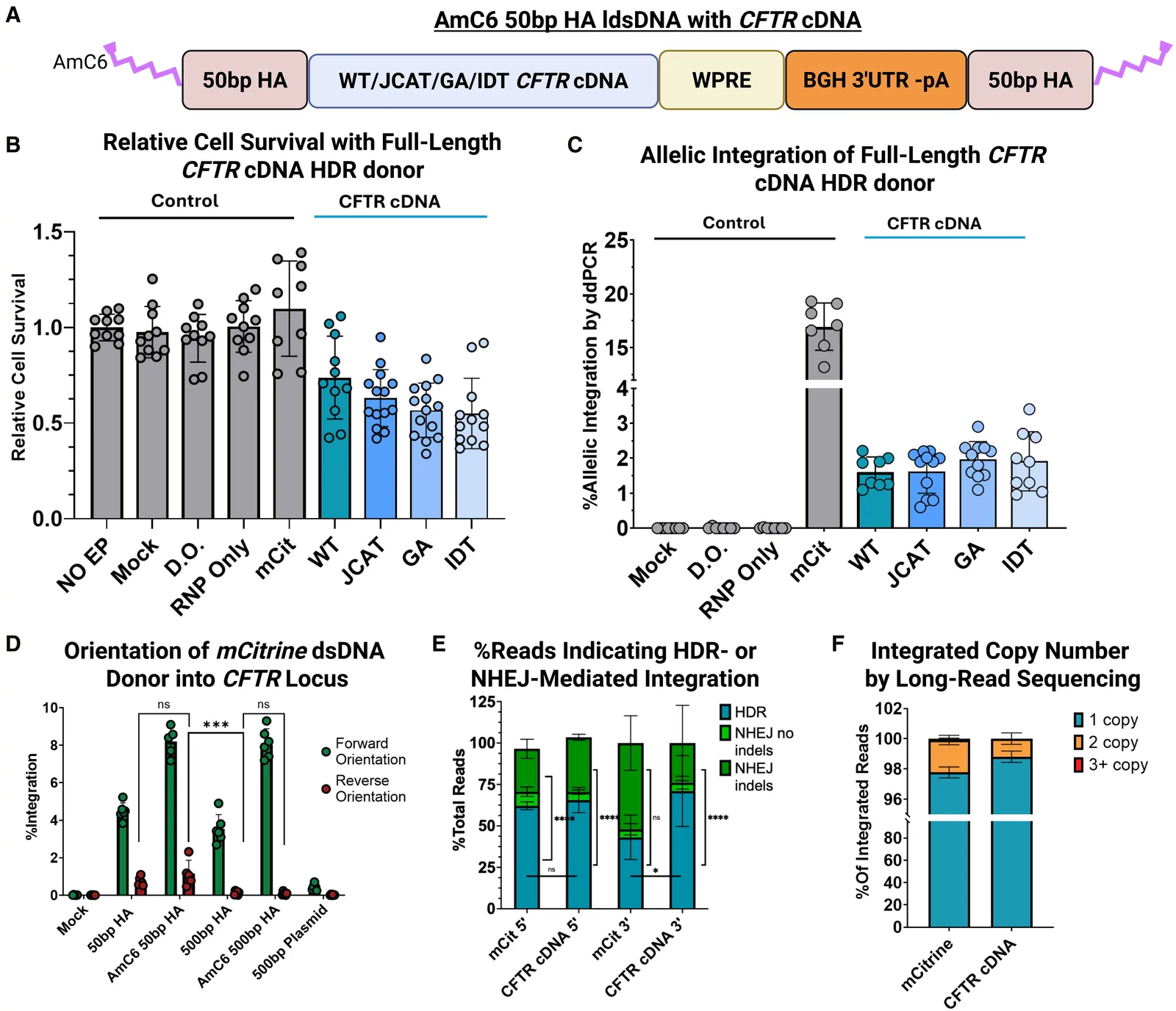
Characterization of editing with *CFTR* cDNA in 16HBEgeG542X (A) Schematic of CFTR ldsDNA donors. (B and C) (B) Relative cell survival and (C) integration of 16HBEgeG542X cells after electroporation delivering AmC6 50 bp HA HDR donors carrying the wildtype and 3 codon optimized *CFTR* cDNA in presence of AZD7648 and ART558 (*n* = 3). (D) Orientation of *mCitrine* transgene across 5 donor types determined *via* ddPCR. (E) Results of next-generation sequencing at the 5′ and 3′ transgene-genome junction of the AmC6 50 bp *mCitrine* or GA codon optimized *CFTR* cDNA donor. (F) Transgene copy number within reads from long-read sequencing across the integrated transgene. Statistical significance was analyzed using a one-way ANOVA followed by multiple paired comparisons for normally distributed data (Tukey test). All statistical tests were two-tailed and ns *p* value of >0.05, ∗*p* < 0.05, ∗∗*p* < 0.01, ∗∗∗*p* < 0.001, ∗∗∗∗*p* < 0.0001. (A) is created with BioRender.

With the optimal editing HDR delivery system identified, the mCitrine cDNA was exchanged for the full-length *CFTR* cDNA. This construct included AmC6-modified homology arms of 50 bp, Woodchuck posttranscriptional response element (WPRE), and bovine growth hormone (BGH) polyadenylation signal sequence. Codon optimization, the process of altering synonymous codons to better align with host-specific expression preferences, can improve translation efficiency, mRNA stability, and protein yield when designing candidate constructs; however, algorithms differ in their approaches, thus potentially affecting downstream expression and function.^33^^,^^34^^,^^35^ Three codon-optimized *CFTR* constructs were therefore designed using the publicly available Java Codon Adaptation Tool (JCAT),^36^ GeneArt (GA),^37^ and IDT algorithms (Figure 4A). These donors were evaluated and compared in the 16HBEge-G542X cell line.^38^ This cell line, created by the Cystic Fibrosis Foundation, has the G542X *CFTR* mutation knocked into the endogenous site. This early stop codon mutation causes transcribed mRNA to be degraded through the nonsense-mediated decay pathway, resulting in a lack of CFTR protein production.

Cells edited with the *CFTR* cDNA donors had significantly increased toxicity compared to the mCitrine donor (Figure 4B). This higher cytotoxicity was attributed to the increased size of the *CFTR* donors, as it is known that larger physical sizes of DNA constructs are correlated to increased specific toxicity.^39^ Integration frequencies of the larger donors significantly dropped as well, compared to the smaller mCitrine donors, with the average integration for each construct ranging between 1% and 2% (Figure 4C).

Since further optimization is largely reliant on improving knock-in efficiency, a greater understanding of the pathway in which the donor is integrating could direct future improvements to the approach. Though the donor was designed for precise HDR insertion, integration can also occur through the homology-independent trapping of the ldsDNA donor via the NHEJ pathway.^40^ Notably, NHEJ-mediated integration can result in the bidirectional insertion of the DNA donor. As such, the orientation of integration was assessed across the five mCitrine donor variants by in-out ddPCR. The ldsDNA donors with 50 bp HAs demonstrated reverse oriented integration, though significantly less than forward integration. There was no detectable reverse orientation with the 500 bp HAs, suggesting a larger HDR-driven mechanism. Finally, the significant increase in forward oriented integration with the AmC6 modification was not accompanied by a similar increase in reverse oriented integration, suggesting the boost occurred through a mechanism distinct from NHEJ (Figure 4D).

Deeper investigation by next-generation sequencing of the 3′ integration junction in 16HBEs edited with the final AmC6 50 bp HA donor allowed for the quantification of HDR- or NHEJ-mediated integration events. HDR reads were binned as scarless integration events, while NHEJ reads were binned as either integration with indels or a doubling of the HAs without indels. Surprisingly, with the smaller *mCitrine* donor, HDR occurred in 43% of total reads, while the GA codon optimized *CFTR* cDNA donor had significantly higher proportion of HDR at 71% of reads. Interestingly, there was no significant difference at the 5′ integration junction (Figure 4E). Finally, because it has been described that viral HDR donors lead to concatemeric integration, the full transgene incorporated into the 5′UTR was sequenced by long-read sequencing.^41^ The number of transgene copies was then analyzed for both donors. The most prevalent sequence correctly contained one copy, though 1%–2% of transgenes contained two copies (Figure 4F).

**Figure 5:**
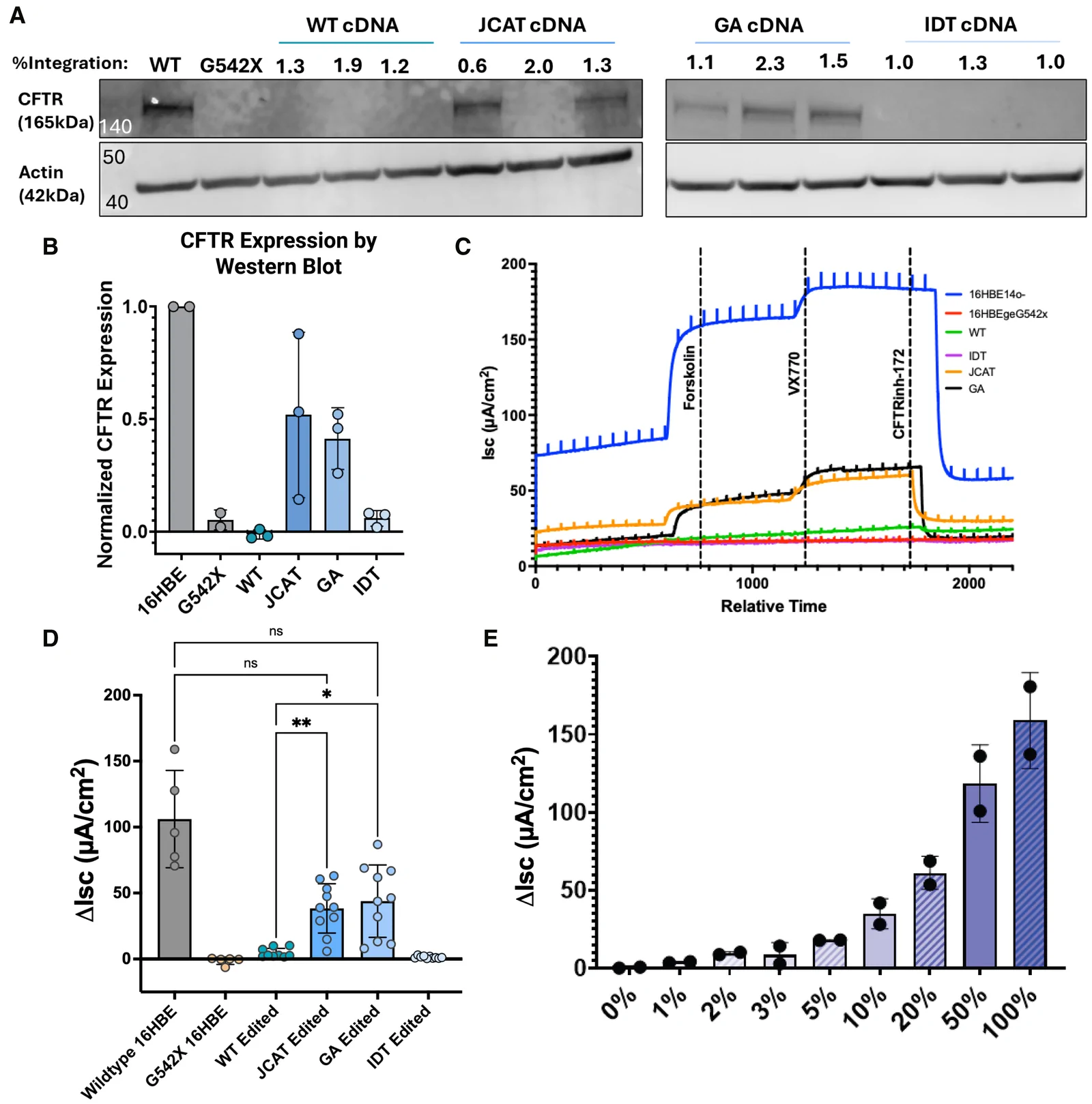
Editing with *CFTR* cDNA in achieves expression and function (A) Western blot from lysate collected from wild type, G542X, and bulk-edited cell population. Percent integration is given below the HDR donor name. (B) CFTR protein levels were quantified by densitometry using ImageJ and normalized to actin levels (*n* = 3). (C) Representative Ussing chamber traces. (D and E) Maximum inhibitable transepithelial chloride current values measured in (D) edited 16HBEge-G542X monolayers or (E) mixed populations of wild type and G542X 16HBE cells at increasing percentages. Statistical significance was analyzed using a one-way ANOVA followed by multiple paired comparisons for normally distributed data (Tukey test). All statistical tests were two-tailed and ns *p* value of >0.05, ∗*p* < 0.05, ∗∗*p* < 0.01, ∗∗∗*p* < 0.001, ∗∗∗∗*p* < 0.0001.

CFTR protein expression from the bulk-edited populations was then assessed via western blot (Figure 5A). Despite the low integration of the *CFTR* constructs, the bulk-edited cell populations with the JCAT and GA codon optimizations achieved roughly 52% and 41% of the CFTR protein expression as in the WT 16HBE control, respectively (Figure 5B). No measurable expression of CFTR protein was seen for the WT or IDT codon optimized *CFTR* cDNA construct, demonstrating the value of codon optimization in increasing translation of the transgene protein, especially when considering events of low integration. Overall, although JCAT yielded slightly higher mean protein expression, the GA construct achieved comparable expression with greater functional recovery, motivating its selection for downstream work.

The bulk-edited 16HBEge-G542X cell populations were seeded in Transwell plates and cultured for 7 days prior to transepithelial chloride current assessment using an Ussing chamber assay to verify functional recovery of the CFTR protein (Figure 5C). The populations edited with the JCAT and GA HDR donors had an average CFTR-inhibitor172-specific current response of −38.4μA/cm^2^ and −43.9μA/cm^2^, respectfully (Figure 5D). Both currents were significantly greater than the cell populations edited with the WT HDR donor (Figure 5D), thereby achieving functional current recovery to 40% of that of WT levels.

Considering the partial functional recovery, it was imperative to confirm that the functional activity from 16HBEge-G542X correlates with functional activity from WT 16HBE cells. Therefore, an Ussing chamber assay was performed on monolayers of different ratios of WT 16HBE:16HBEge-G542X. We observed that CFTR function in each monolayer correlated with the percentage of wild-type cells. Importantly, previous studies have found that a population of just 10% CFTR-expressing cells could achieve wild-type levels of function.^23^ We found that integration rates of roughly 2% with the *CFTR* transgene-constructs achieved CFTR-specific inhibited currents at levels between the wildtype:G542X monolayers of 10% and 20% wild-type 16HBEs (Figure 5E).

## Discussion

Broad categories of nucleic acid-based gene therapies, including antisense oligonucleotide (ASO), tRNA, mRNA, lentiviral vectors, and DNA-based therapeutics are of great interest for the treatment of CF and are currently under rapid advancement.^42^^,^^43^ However, there are limitations associated with these approaches, including but not limited to: (1) such approaches often only target a single mutations class; (2) lentiviral vector, though able to carry large cassettes are unable to target ABSCs as VSVG targets LDL receptors on the basolateral, but not apical side; (3) AAVs have limited packaging capacities and repeated dosing is largely inefficient; (4) AAVs trigger immune responses; and (5) common delivery mechanisms must penetrate the thick mucus layer and more.^44^ Despite these limitations, it is crucial that a continued effort is made as gene editing of long-lived stem cell niches within affected tissues holds promise for sustained and robust treatment for CF patients. Recently, exciting data on platforms that leverage base editors or CRISPR-based correction of small mutations using single-stranded oligodeoxynucleotide (ssODN) donors have been published.^45^^,^^46^ However, these approaches require the design and validation of reagents customized to address single mutations.

With this understanding, the primary goal of this work was to develop a genome-editing approach using nonviral reagents that could functionally and universally correct all CF-causing mutations and could be adapted for nanoparticle-based delivery. A candidate DNA HDR construct was designed to maximize the editing and functional efficacy of the constructs with an aim of overcoming nanoparticle-based delivery bottlenecks and ensuring straightforward encapsulation.^47^^,^^48^^,^^49^^,^^50^^,^^51^ Ultimately, an AmC6 chemically modified ldsDNA with 50 bp HA proved to be the most effective HDR donor. Additionally, the shorter homology arms enhance the approach’s safety profile, as it excludes the *CFTR* promoter in order to prevent local dysregulation of genes in the event of random off-target integration. This donor design could be utilized more broadly for other diseases characterized by mutations in large genes exceeding the packaging limitation of AAV or research that requires a nonviral HDR donor for targeted site-specific integration.

The decreased integration efficiency of the full-length *CFTR* cDNA constructs demonstrate that further optimization of the strategy is necessary to achieve a higher proportion of allele integration events in the cell line population. Further optimization of these editing reagents to increase efficiency will be important when moving toward the less HDR-prone primary ABSCs.^52^^,^^53^ This obstacle could partially be overcome by using LNPs rather than electroporation to deliver the editing reagents as LNPs have been demonstrated to have decreased cytotoxicity and induce fewer global changes to the transcriptome.^54^

We have minimized the size of the donor constructs to enable straightforward integration with nanoparticle-based delivery systems; however, the size of these constructs still exceeds the size of DNA-based cargoes that have previously been delivered using LNPs. To bridge this gap, our team has parallel work recently conducted in which an LNP platform was optimized with the capability to package and deliver these large ldsDNA constructs to the 16HBE cell line. Editing efficiencies modestly improved, but more encouragingly, expression and function equivalent to wild-type controls was achieved.^55^ While we have demonstrated that the use of AZD7648 and ART558 can increase editing rates, the application and use of these small molecules for *in vivo* LNP-based delivery as a co-encapsulating system requires optimization, safety, and feasibility studies.

However, delivery to primary ABSCs remains a major hurdle to *in vivo* treatment.^45^ For instance, administration of LNPs *via* aerosolization or intravenous injection requires that nanocarriers traverse either the thick mucus layer, epithelial barrier, or endothelial barrier.^56^^,^^57^ Similar *ex vivo* strategies, namely autologous transplantation of edited ABSCs, remain an unsolved challenge despite encouraging recent results.^58^ Still, several exciting developments toward optimizing editing and delivery reagents are proving essential toward eventual clinical translation. Fortunately, with the reagents described and their compatibility with LNP-based delivery, this modular system can be tuned as necessary to address these challenges without altering the gene-editing strategy.

Airway cell populations containing just 2% edited alleles restored approximately 40% of the functional activity of wild-type cells, which supports the field’s observations that low rates of cellular correction can yield therapeutically meaningful correction of the CF phenotype.^23^ Notably, our flow cytometry validation of the mCitrine reporter showed mCitrine^+^ cells at roughly twice the allelic integration rate measured by ddPCR, consistent with predominantly monoallelic integration. This indicates that ddPCR is not substantially underestimating integration, therefore the disproportionate functional recovery reflects the established threshold whereby a low percentage of corrected cells restores near-physiological CFTR-dependent current. Furthermore, sequencing of the transgene insertion site proved informative when determining orientation and correction pathways of ldsDNA constructs. As mentioned, codon-optimization techniques and BGH and WPRE sequences were included for their well-known capacity to boost expression, highlighting the necessity of regulatory elements in transgene design.^59^^,^^60^^,^^61^^,^^62^^,^^63^^,^^64^^,^^65^^,^^66^

Together, these results mark an important step toward a universal gene-editing strategy to functionally replace the mutant *CFTR* gene. This research lays the foundation for a single, off-the-shelf therapy for virtually all CF patients, potentially offering hope to patients with currently untreatable mutations.

## Materials and methods

### Cell culture

T84 cells (ATCC; cat: CCL-248 Manassas, VA) were cultured in F-12K Hams Medium (ATCC; cat: 30-2004), with 10% fetal bovine serum (Omega Scientific FB-02 Tarzana, CA), and 1% penicillin/streptomycin/glutamate (Thermo Fisher Scientific; cat: 10378016, Grand Island, NY). Passages were carried out weekly by detaching cells using 0.25% trypsin in EDTA (Thermo Fisher Scientific; cat: 25200114).

16HBE14o- cells were acquired from Millipore Sigma. 16HBEge-G542x cells were obtained via a materials transfer agreement between the Regents of the University of California, Los Angeles and Dr. Hillary Valley at the Cystic Fibrosis Foundation (CFF). 16HBE cell lines were cultured in Eagle’s Minimum Essential Medium (ATCC; cat: 30-2003) with 10% fetal bovine serum (Omega Scientific; cat: FB-02), 1% penicillin/streptomycin/glutamate (Thermo Fisher Scientific; cat: 10378016), herein referred to as E10. Cells were cultured in flasks or well plates that were pre-coated with fetal bovine serum albumin (Millipore Sigma; cat: A7979 Saint Louis, MO), bovine collagen solution (Advanced Biomatrix; cat: 5005 Carlsbad, CA), and fibronectin from human plasma (Thermo Fisher Scientific; cat: 33016015). Passages were carried out weekly by detaching cells using 0.25% trypsin in EDTA, quenching the reaction with E10, and splitting between 1:2 and 1:10.

### Donor template design and generation

Double-stranded DNA (dsDNA) donor synthesis and amplification: dsDNA donor fragments were synthesized as gBlocks by Integrated DNA Technologies (Coralville, IA). These gBlocks were subsequently cloned into plasmids using the TOPO Zero Blunt Cloning Kit (Thermo Fisher Scientific; cat: K2800J10). The dsDNA donors were then amplified by PCR from these plasmids with Platinum SuperFi II DNA Polymerase using the manufacturer’s protocol (Thermo Fisher Scientific; cat: 12369010). Amplification utilized oligonucleotide primers that were modified at the 5′ end with AmC6, also supplied by Integrated DNA Technologies. The PCR products were then purified using 1X SPRI paramagnetic bead-based purification (Ampure XP; Beckman Coulter; cat: A63881 San Jose, CA). Following the first round of purification, the DNA was eluted twice with 150 μL 1/10 TE in water. The 300 μL total product was digested with Dpn1 at 37 °C for 1 h to remove residual plasmid DNA. This was followed by a second round of purification, again with 1× SPRI beads. The final product was eluted in 80 μL of pure TE.

### Delivery of RNP and HDR donor

T84 cells were electroporated when cultures were 70%–80% confluent. The day prior to transfection, 16HBE cells were split 1:2. For experiments with the full-length *CFTR* cDNA donors, 16HBE cells were cultured in 10 μM Y-27632 ROCK inhibitor (StemCell Technologies; cat: 72304 Vancouver, Canada) starting from this split for the remainder of the experiment. 100 pmol of purified Cas9-NLS protein (MacroLab, UC Berkeley, CA) was added to 120 pmol sgRNA (Synthego Corporation, Menlo Park, CA) and let sit at room temperature for 20 min to form an RNP complex. Cells were electroporated with the Lonza 4D-Nucleofector System (program FI 115 for T84s and CM 137 for 16HBEs) in SF Cell Line solution at a density of 200,000 cells per 20 μL reaction with RNP, electroporation enhancer (IDT; cat: 1075916), and the HDR donor. Following electroporation, cells were let rest for 10 min then added to recovery plate with E10 medium and, if specified, 0.5 μM AZD7648 (Selleckchem; cat: S8843 Houston, TX) and 0.5, 1, 2, or 3 μM ART558 (MedChem Express; cat: HY-141520 Monmouth Junction, NJ) at various concentrations highlighted in the appropriate Figures.

### MTT assay

Immediately after electroporation, 100 μL of the 500 μL of cells was seeded in a 96 well plate. 24 h later, 10 μL of MTT reagent (Biotium; cat: 30006 Fremont, CA) was added and incubated for 4 h at 37 °C. Then, the formazan product was solubilized by adding 200 μL DMSO and quantified by spectrometry at wavelength 570 nm. Background was measured at wavelength at 630 nm and subtracted from the on-target readings. Relative cell viability was normalized to the non-electroporated controls.

### Integration analysis

Genomic DNA was extracted from edited cells for integration site analysis using the Invitrogen PureLink Genomic DNA Kit (Thermo fisher Scientific; cat: K182002) and quantified using the NanoDrop system (Thermo Fisher Scientific; cat: ND-2000). DNA samples were then analyzed by ddPCR to measure integration rates. Two sets of primers were duplexed, each with their own fluorescent probe (FAM/HEX, Table S2). To measure integration rates of the mCitrine reporter cassette, one primer was complementary to a *CFTR* gene sequence upstream from the left homology arm of the donor. The second primer bound to the mCitrine reporter cassette to allow for specific measurement of the integrated mCitrine transgene distinct from the endogenous *CFTR* gene. To measure integration rates of the *CFTR* donor cassette, one primer was bound to the BGH polyA signal sequence to enable specific measurement of the integrated *CFTR* donor. The second primer was complementary to a *CFTR* gene sequence downstream of the right homology arm of the donor cassette. The FAM-conjugated nucleotide probe with a quencher also bound to the minus-strand DNA near the second primer. A reference primer/probe set was also delivered to recognize the *SDC4* gene on chromosome 20. 1 μL of DraI endonuclease (New England Biolabs; cat: R0129S Ipswich, MA) was added to the reaction mixture (an enzyme that does not disrupt the experimental or reference amplicons) to reduce background. Each sample was digested at 37 °C for 1 h before droplet generation with the Bio-Rad QX200 Droplet Generator (Bio-Rad; cat: 186-4002 Irvine, CA). The prepared samples were then assayed via the QX200 Bio-Rad Droplet Reader on the “Absolute” measurement setting (Bio-Rad; Cat: 186-40).

To assess NHEJ- versus HDR-mediated integration, in-out PCR was performed on genomic DNA from edited 16HBE cells with primers “CAGCCATCTGTTGTTTGCC” and “CCAACCCATACACACGCC” for the 3′ transgene junction with the Illumina R1/R2 adapters added to the 5′ end. For the 5′ junction, a common primer to binding to the *CFTR* locus “AAGGAAGGGGTGGTGTG” was used with donor-specific reverse primers (“cgaccaggatgggcac” for mCitrine and “TAGGCCGGGTCCAGCTGAAGA” for the *CFTR* cDNA). The amplicon was purified using 1.2× AmpureXP SPRI beads and sequenced using Quintara Bioscience’s AmpExp Amplicon NGS service and analyzed by quantifying read abundance (Quintara Biosciences, Boston, MA).

Concatemeric integration was assessed by PCR using primers that flank the ldsDNA homology arms (primers “CCAACCCATACACACGCC” and “AAGGAAGGGGTGGTGTG”). An extension time capable of amplifying 3 integrated copies was selected for each donor. The amplicon was purified using 0.5× AmpureXP SPRI beads to select for amplicons greater than 500 bp to predominantly isolate amplicons with the integrated transgene. The purified amplicons were nanopore sequenced (Plasmidasaurus, San Francisco, CA) and analyzed using a custom Python script to bin reads based on transgene copy number.

### Cutting efficiency

The Cas9 cutting efficiency was measured via Inference of CRISPR Edits (ICE). PCR on genomic DNA extracted from edited cell populations was used to generate a 728 bp amplicon for the 5′UTR guides (primer sequences: “AAAGCCGCTAGAGCAAATTT,” “TGTTGGCTGAATTCAGTCAA”) and 700 bp for the intron 1 guides (primer sequences: “GTCTGACAATTCCAGGCGCT,” “TTATTGATGCCTAGAGGGCAGA”). The resulting amplicon was Sanger sequenced and analyzed with the Synthego ICE web tool (Synthego Corporation, Menlo Park, CA).

### Off-target analysis

dsDNA breaks at off-target cut sites were identified using genome-wide, unbiased identification of DSBs enabled by sequencing (GUIDE-seq) using the protocol described by Malinin et al.^24^^,^^25^ 16HBE or T84 cells were electroporated with Cas9-sgRNA RNP targeting the *CFTR* locus followed by the addition of 3 μM or 5 μM GUIDE-seq oligo. Sites of oligo trapping were enriched by locus-specific PCR followed by a second round of PCR to add index and adapter sequences necessary for next-generation sequencing. The amplicons were normalized using densitometry and pooled. The final library was quantified using ddPCR and sequenced using Illumina MiSeq. Data was analyzed following the bioinformatics pipeline from Malinin et al.^24^^,^^25^

The nominated site at *ST6GAL2* was amplified using primers “aggggccttggtcggttgtg” and “agactccttgaggttaagaatcca” with the Illumina R1/R2 adapters added to the 5′ end. The amplicon was purified using 1.2× AmpureXP SPRI beads and sequenced using Quintara Bioscience’s AmpExp Amplicon NGS service (Quintara Biosciences, Boston, MA).

### Western blot

For immunoblots, cells were lysed in RIPA Lysis and Extraction Buffer (Thermo Fisher Scientific; cat: 89901 Grand Island, NY) with added HALT protease inhibitor (Thermo Fisher Scientific; cat: 87786 Grand Island, NY) at a 1× concentration following the manufacturer’s protocols. Lysate concentrations were determined using the Pierce BCA protein assay (Thermo Fisher Scientific; cat: 23227 Grand Island, NY) following the manufacturer’s protocol. Samples were treated for sodium dodecyl sulfate-polyacrylamide gel electrophoresis (SDS-PAGE) with NuPAGE LDS Sample Buffer (Thermo Fisher Scientific; cat: NP0007) and NuPAGE Sample Reducing Agent (Thermo Fisher Scientific; cat: NP0009), each to a 1× concentration. Lysates were diluted to contain 50 μg of total protein for immunoblot gel loading to keep the total amount of protein loaded per lane constant to allow for valid loading controls. Note, CFTR protein becomes insoluble if the sample is heated above 60 C and will not enter the stacking gel. As such, the samples were denatured at 37 C for 20 min prior to loading into the stacking gel. Wild-type cells 16HBE14o- were used as a control to indicate the relative expression levels of CFTR protein. CFTR levels were detected using Ab UNC-596 (from J. Riordan, UNC^67^) at 1:500 in 5% milk in TBST. Protein quantification was assessed through densitometry via the ImageJ software. CFTR protein levels were normalized to the actin protein levels after quantification.

### Ussing chamber assay

16HBEge-G542X cells were seeded on 12 mm polyester SnapWell inserts (Corning; cat: 3801 Corning, NY) coated in collagen type IV from human placenta (Millipore Sigma; cat: C5533) at a density of 500,000 cells/well and cultured in E10 in basolateral and apical chambers for at least 7 days prior to Ussing assays. Assays were performed using an EasyMount Ussing chamber (Physiologic Instruments) at 37°C in HEPES buffered solutions with an imposed chloride gradient across the epithelia. The basolateral solution contained (mM): 137 NaCl, 4 KCl, 1.8 CaCl2, 1 MgCl2, 10 HEPES, and D-Glucose, adjusted to pH 7.4 with NaOH/HCl ([Cl−]total: 146.6 mM). The apical solution was matched to the basolateral except for (mM): 137 Na-gluconate replaced 137 NaCl ([Cl−]total: 9.6 mM).

Electrode tips (physiologic instruments) were partially filled with 3% LB Agar (Thermo Fisher Scientific; cat: 22700041) in 3M KCl and backfilled with 3M KCl.

Cells grown on SnapWell inserts were mounted in the chambers and allowed to equilibrate for 10–15 min before recording baseline short-circuit current (Isc) for 10 min. At 10 min intervals, 10 μM amiloride, 10 μM forskolin, 1 μM VX-770, and 20 μM CFTR-Inh172 were added sequentially to the apical and basolateral side of the chamber. At 10 min after the initial dose of CFTR-Inh172, a second dose of 20 μM CFTR-Inh172 was added to the basal and apical chambers to extinguish any remaining current, and, 5 min later, 100 μM adenosine triphosphate (ATP) was added to the basolateral and apical chambers which served as a quality control for the activation of calcium-activated Cl currents. I_sc_ was recorded for an additional 10 min before ending the experiment.

## Data and code availability

All data are provided in the main text or supplemental information. Raw sequencing data from GUIDE-seq and next-generation sequencing can be made available upon request.

## Acknowledgments

We thank the Cystic Fibrosis Foundation (CFF) for creating and generously sharing the 16HBEge-G542X cell line and for the CFTR Antibodies Distribution Program from which we received the UNC-596P CFTR antibody. We also thank 10.13039/100000900California Institute for Regenerative Medicine Grant DISCO0-14458 (B.N.G., D.B.K., S.J.J.), the CFF Grant JONAS20XX0 (S.J.J., D.B.K., B.N.G.), and a New Horizons Award from the 10.13039/100016616Cystic Fibrosis Research Institute, award #: 20213480 (S.J.J., B.N.G., D.B.K.) for providing the funds for this project. Figures created in BioRender. Hollis, R. (2026) https://BioRender.com/b9ogfke.

## Author contributions

V.S., P.G.A., B.N.G., S.J.J., and D.B.K. conceived the study. V.S., P.G.A., C.J.J., L.E.L., R.A.F., J.D.L., B.I., E.C.D., N.R.F., B.N.G., S.J.J., and D.B.K. developed methodology, performed the experiments, and interpreted the data. The manuscript was drafted by V.S. and C.J.J. and reviewed and edited by V.S., C.J.J., P.G.A., L.E.L., R.A.F., J.D.L., R.A.S., B.I., E.C.D., N.R.F., B.N.G., S.J.J., and D.B.K.

## Declaration of interests

P.G.A., R.A.F., B.N.G., D.B.K., and S.J.J. are inventors on US patent applications filed by the Regents of the University of California relating to the design of site-specific *CFTR* gene-editing machinery.

## Supplemental information

Document S1. Figures S1–S3, Tables S1, and S2

Document S2. Article plus supplemental information
