# Supplementary material for: Double-stranded DNA donors and CRISPR-Cas9 for universal correction of mutations causing cystic fibrosis in human airway cells
Source: Mol Ther Nucleic Acids. 2026 Aug 3;37(3):103049. doi: 10.1016/j.omtn.2026.103049 (PMC13521165; doi:10.1016/j.omtn.2026.103049)
Supplement: Supplementary file 1 [file mmc1.pdf]

OMTN, Volume *37*

Supplemental information

Double-stranded DNA donors and CRISPR-Cas9 for universal correction of mutations causing cystic fibrosis in human airway cells

Vrishti Sinha, Paul G. Ayoub, Colin J. Juett, Lindsay E. Lathrop, Ruth A. Foley, Ruby A. Sims, Joseph D. Long, Emily C. Duggan, Neil R. Fernandes, Beate Illek, Brigitte N. Gomperts, Steven J. Jonas, and Donald B. Kohn

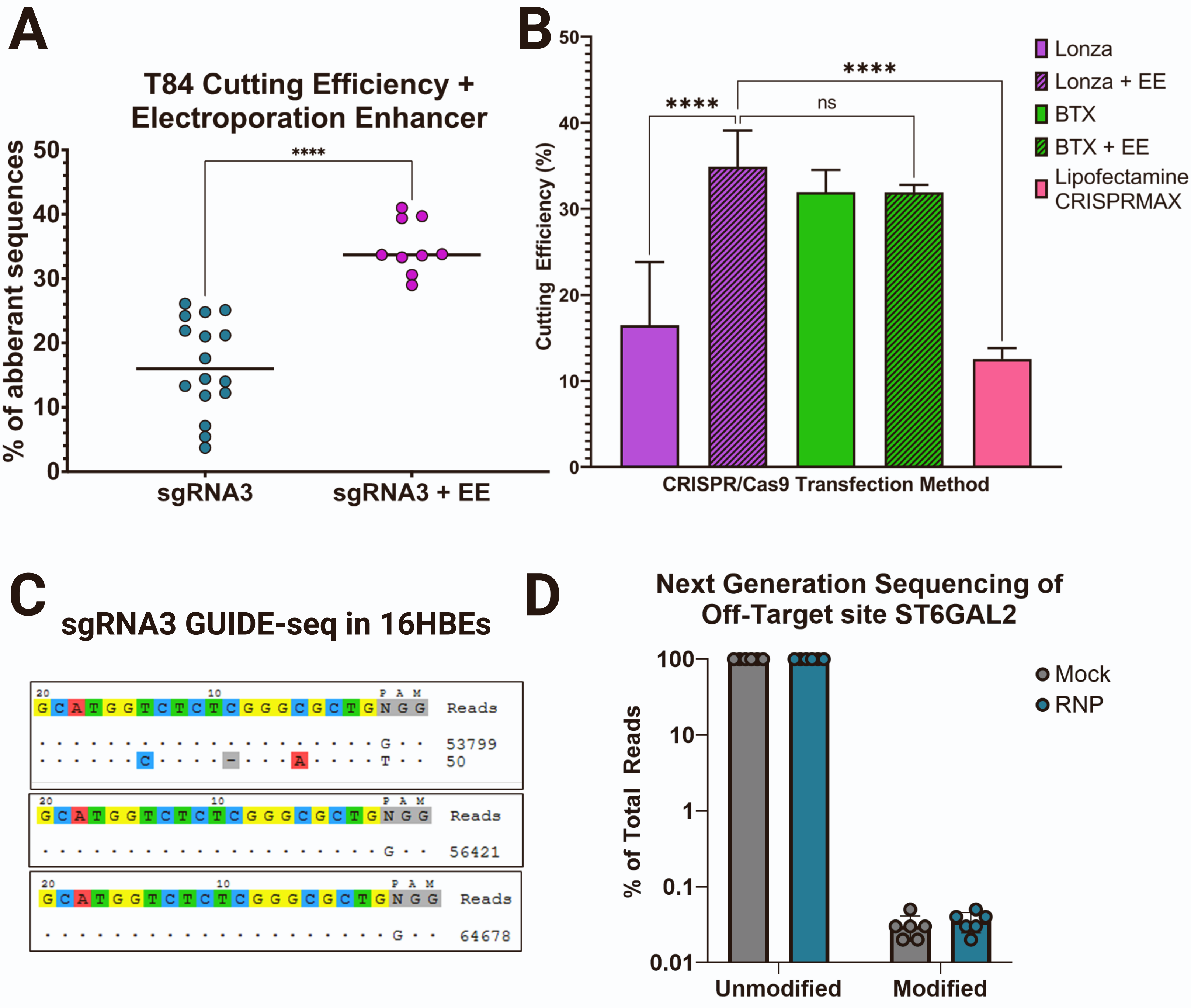
A
T84 Cutting Efficiency +
Electroporation Enhancer
% of abberant sequences
0
10
20
30
40
50
****
sgRNA3
sgRNA3 + EE
B
Cutting Efficiency (%)
0
10
20
30
40
50
****
ns
****
CRISPR/Cas9 Transfection Method
Lonza
Lonza + EE
BTX
BTX + EE
Lipofectamine
CRISPRMAX
C
sgRNA3 GUIDE-seq in 16HBEs
20
10
P A M
G C A T G G T C T C T C G G G C G C T G N G G
Reads
53799
C
-
A
T
50
56421
64678
D
Next Generation Sequencing of
Off-Target site ST6GAL2
% of Total Reads
100
10
1
0.1
0.01
Unmodified
Modified
Mock
RNP

Figure S1. Electroporation Enhancer Improves Delivery of sgRNA-Cas9 RNP

(A) Cutting efficiency, measured by %indels, in T84 cells with or without electroporation enhancer (n=6). Each dot represents a different electroporated replicate (n=3). (B) Cutting efficiency following delivery through different platforms. Data is presented as means ± standard deviation. (C) Results from GUIDE-seq analysis of sgRNA3 in 16HBEs from the highest dose of dsODN delivered (5µM) in GUIDE-seq analysis (n=3). (D) Results from deep sequencing at nominated off-target site, *ST6GAL2,* in 16HBEs edited with sgRNA3 analyzed using CRISPResso2 (n=6). Statistical significance was analyzed using a One-Way ANOVA followed by multiple paired comparisons for normally distributed data (Tukey test). All statistical tests were two-tailed and a ns p value of > 0.05, *$p < 0.05$, **$p < 0.01$, ***$p < 0.001$, ****$p < 0.0001$.

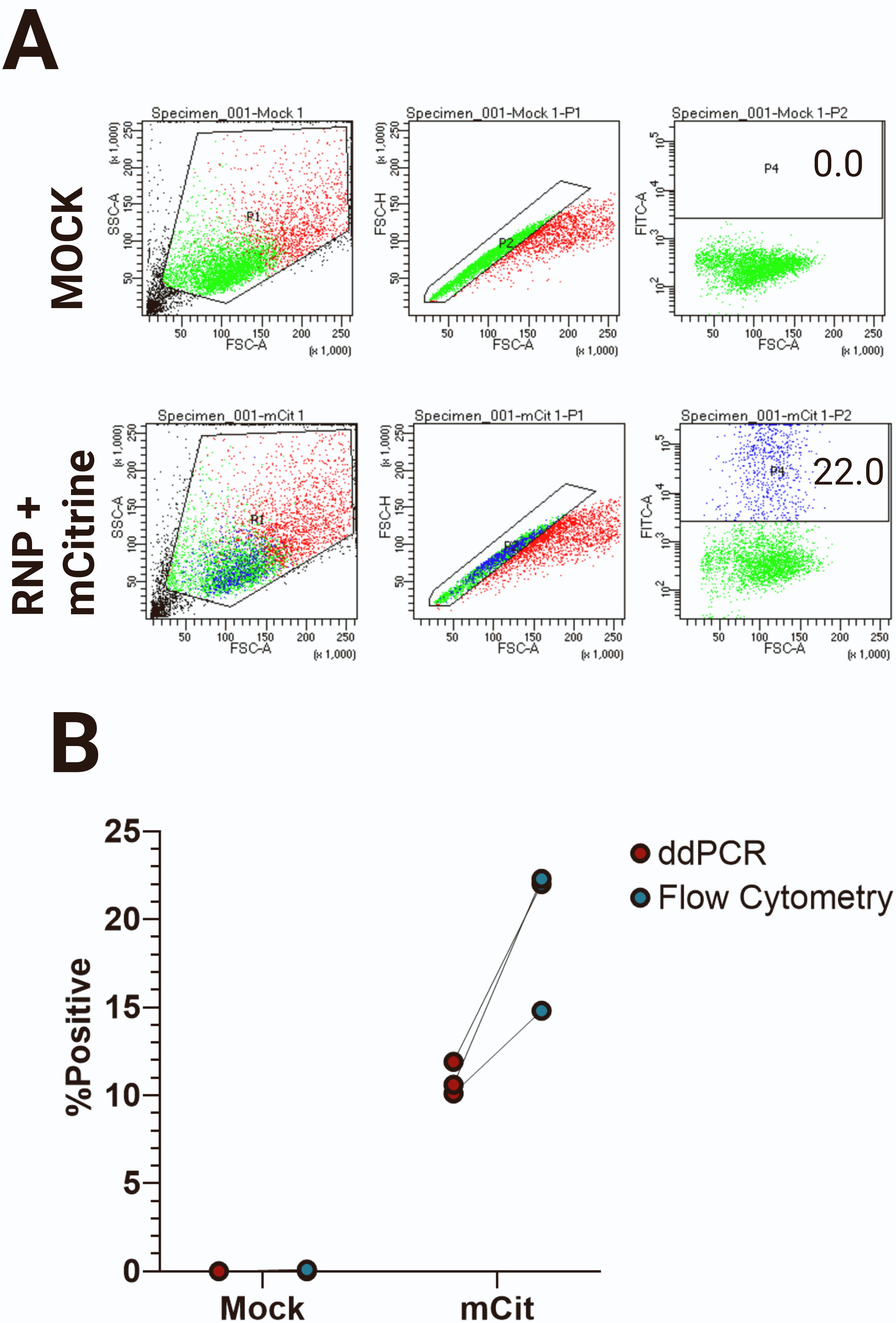
A
MOCK
RNP + mCitrine
Specimen_001-Mock 1
Specimen_001-Mock 1-P1
Specimen_001-Mock 1-P2
Specimen_001-mCit 1
Specimen_001-mCit 1-P1
Specimen_001-mCit 1-P2
SSC-A
FSC-A
FSC-H
FITC-A
(x 1,000)
P1
P2
P4
0.0
22.0
B
%Positive
ddPCR
Flow Cytometry
Mock
mCit

Figure S2. Flow Cytometry Results Compared to ddPCR Quantitfication

(A) Representative flow plots of 16HBE cells 72 hours after editing with RNP and *mCitrine* ldsDNA HDR donor. (B) Quantification of mCitrine^{+} cells (blue) or allelic integration (red) of same cells.

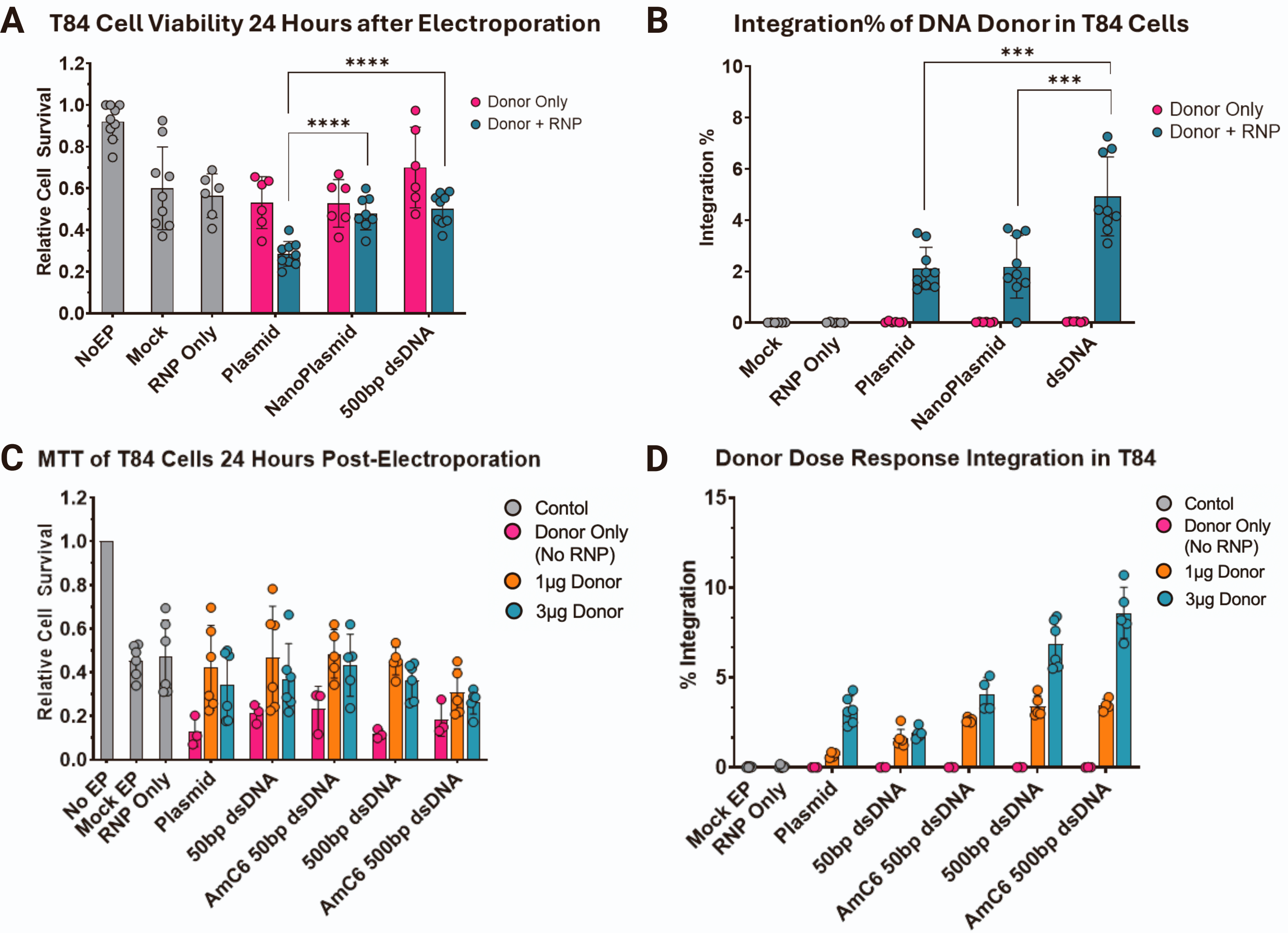

A
T84 Cell Viability 24 Hours after Electroporation
Relative Cell Survival
1.2
1.0
0.8
0.6
0.4
0.2
0.0
****
****
Donor Only
Donor + RNP
NoEP
Mock
RNP Only
Plasmid
NanoPlasmid
500bp dsDNA
B
Integration% of DNA Donor in T84 Cells
Integration %
10
8
6
4
2
0
***
***
Donor Only
Donor + RNP
Mock
RNP Only
Plasmid
NanoPlasmid
dsDNA
C
MTT of T84 Cells 24 Hours Post-Electroporation
Relative Cell Survival
1.2
1.0
0.8
0.6
0.4
0.2
0.0
Contol
Donor Only
(No RNP)
1µg Donor
3µg Donor
No EP
Mock EP
RNP Only
Plasmid
50bp dsDNA
AmC6 50bp dsDNA
500bp dsDNA
AmC6 500bp dsDNA
D
Donor Dose Response Integration in T84
% Integration
15
10
5
0
Contol
Donor Only
(No RNP)
1µg Donor
3µg Donor
Mock EP
RNP Only
Plasmid
50bp dsDNA
AmC6 50bp dsDNA
500bp dsDNA
AmC6 500bp dsDNA

Figure S3. Optimization of HDR Donors in the T84 Cell Line

(A) Relative cell survival and (B) integration after electroporation delivering plasmid, nanoplasmid, or linear dsDNA HDR donor with 500bp HA construct to T84 cells (n=3). (C) Relative cell survival and (D) integration after electroporation delivering 50bp homology arm donors with or without AmC6 and 500bp donors with or without AmC6 to T84 cells (n=3). Statistical significance was analyzed using a One-Way ANOVA followed by multiple paired comparisons for normally distributed data (Tukey test). All statistical tests were two-tailed and a ns p value of > 0.05, *$p < 0.05$, **$p < 0.01$, ***$p < 0.001$, ****$p < 0.0001$.

Table S1

Description of sgRNAs, sequences, and *CFTR* location.

sgRNA Name	Sequence	Location
sgRNA 3	GCATGGTCTCTCGGGCGCTG	5'UTR
sgRNA 13	GCTAGTATATGATTATTTGG	Intron 1
sgRNA 14	CTTTGTCAAAGGGATTGGGA	Intron 1
sgRNA 15	AGTCAATTTCTATAAATACC	Intron 1
sgRNA 17	AAGACACGTGCCCACGAAAG	Intron 1
sgRNA 18	ACACGTGCCCACGAAAGAGG	Intron 1
sgRNA 19	CACGTGCCCACGAAAGAGGA	Intron 1

Table S2

Description of ddPCR primer probe sets for each donor

Donor	Transgene Primers and Probe	Reference Gene Primers and Probe
50bp HA mCitrine	F: AAGGAAGGGGTGGTGTG<br>R: cgaccaggatgggcac<br>P: TCTCTGACCTGCTGTGATGTC	F: AATACGCTGAGTGTCTCTTC<br>R: CTCACTCTCTTCAACGGG<br>P: TGAGAGGGCAGGGTCTGGGA
500bp HA mCitrine	F: GGAGGGTCTAGGAAGCT<br>R: tcgcccttgctcacCAT<br>P: TCTCTGACCTGCTGTGATGTC	F: CTCTTCCAAAATACGCTGAG<br>R: GCAGAAGAATGACTCAATGC<br>P: TCCTCACTCTCTTCAACGGGT
50bp/500bp HA mCitrine reverse orientation	F: GGAGGGTCTAGGAAGCT<br>R: CAGCCATCTGTTGTTTGCC<br>P: TCTCTGACCTGCTGTGATGTC	F: CTCTTCCAAAATACGCTGAG<br>R: GCAGAAGAATGACTCAATGC<br>P: TCCTCACTCTCTTCAACGGGT
50bp HA *CFTR* cDNA	F: CAGCCATCTGTTGTTTGCC<br>R: CCAACCCATACACACGCC<br>P: AGAGCCCACCGCATCCCCAG (FAM)	F: AATACGCTGAGTGTCTCTTC<br>R: CTCACTCTCTTCAACGGG<br>P: CCCACCGAACCCAAGAAACTAGAGGAGAAT (HEX)
